# Coinfection with Giardiasis and Taeniasis

**DOI:** 10.4269/ajtmh.25-0742

**Published:** 2026-04-07

**Authors:** Yu Miyazaki, Akitoshi Ueno, Takuya Adachi

**Affiliations:** Department of Infectious Diseases, Tokyo Metropolitan Toshima Hospital, Tokyo, Japan

A 36-year-old Ethiopian man presented to the study outpatient clinic with a history of loose stools that had lasted for 1 month. He reported noticing 1-millimeter-sized white fragments in his stool on one occasion. He had lived in Ethiopia until 3 months before presenting to the clinic and regularly consumed raw beef. As a Muslim, he had no history of pork consumption. His vital signs and physical examination results were unremarkable. Two stool examinations yielded negative results for helminth eggs but revealed *Giardia* cysts (Figure 1), and he was treated with metronidazole (1,500 mg/day for 7 days). His symptoms initially improved but recurred several weeks later. A repeat stool examination revealed an embryonated Taenia egg (Figure 2A), and he was admitted for treatment. After laxative preparation, he was administered praziquantel (1,200 mg), followed by polyethylene glycol. Subsequently, a 2.5-meter tapeworm lacking a scolex (Figure 2B) was expelled. Histopathology revealed a gravid proglottid densely filled with eggs (Supplemental Figure 1), and a restriction fragment length polymorphism analysis of a polymerase chain reaction-amplified *cox1* fragment helped identify the parasite as *Taenia saginata*. Three months later, the patient remained asymptomatic.

**Figure 1.**
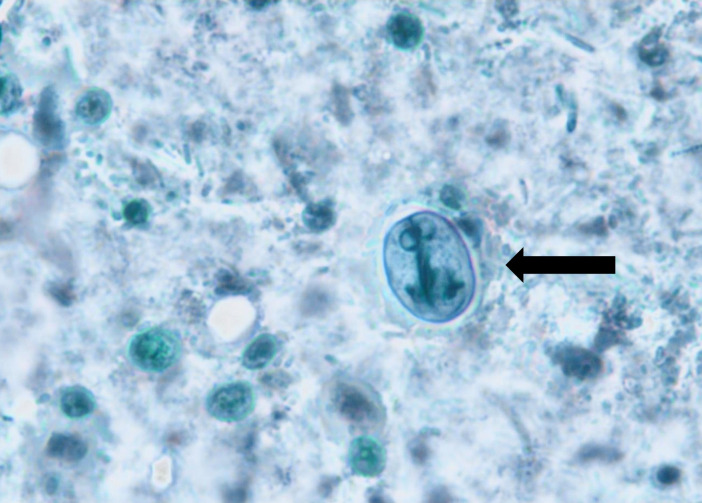
*Giardia* spp. cyst identified in a stool sample using Kohn staining. The staining highlights the typical oval morphology and internal structures, including axonemes and nuclei, aiding in microscopic identification. (original magnification: 400×).

**Figure 2.**
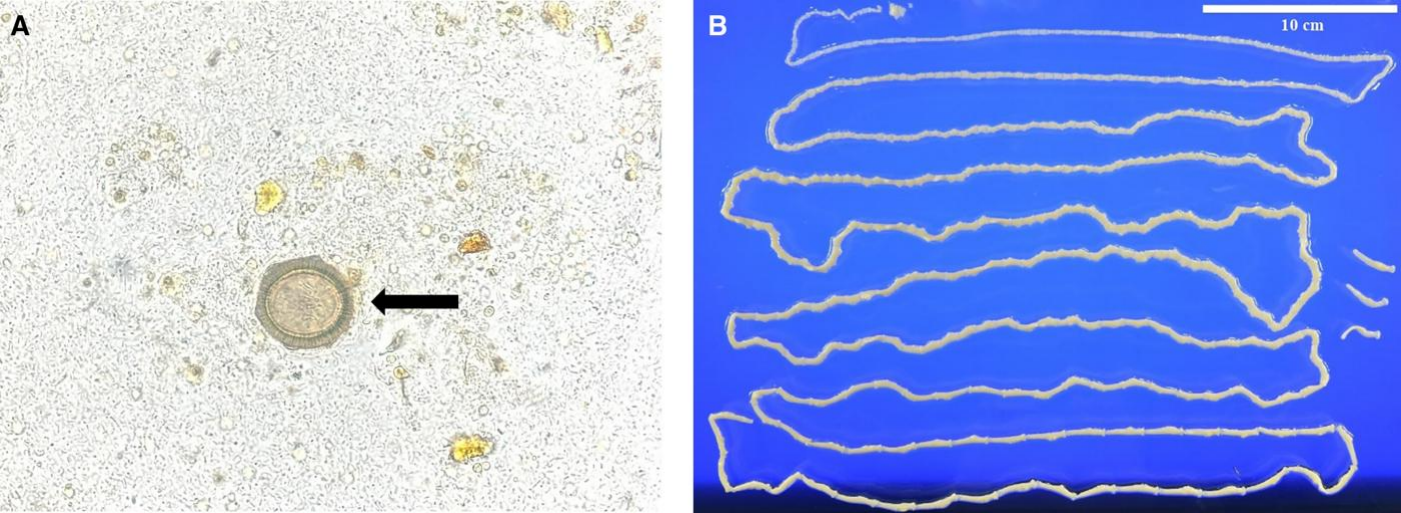
Stool and parasite specimens of *Taenia saginata* (*T. saginata*). (**A**) Repeat stool examination revealed an embryonated egg containing an oncosphere (original magnification: 400×). (**B**) A *T. saginata* specimen lacking a scolex was expelled from the patient after treatment with praziquantel.

Intestinal parasitic infections are uncommon in high-income countries but remain prevalent worldwide.^1^ They often cause nonspecific gastrointestinal symptoms, making accurate diagnosis difficult. Because a single stool examination may fail to result in the identification of pathogens, performing at least three stool examinations is recommended.^2^ Mixed infections have also been reported,^3^ indicating that gastrointestinal symptoms may involve multiple pathogens.

In the present case, repeated stool examination led to the diagnosis of a giardiasis and taeniasis coinfection, highlighting the importance of repeated testing when parasitic infection is suspected.

## Supplemental Materials

10.4269/ajtmh.25-0742 Supplemental Materials
