# Supplementary material for: Coinfection with Giardiasis and Taeniasis
Source: Am J Trop Med Hyg. 2026 Apr 7;115(1):3–4. doi: 10.4269/ajtmh.25-0742 (PMC13326953; doi:10.4269/ajtmh.25-0742)

Supplemental Figure 1. Hematoxylin–eosin–stained section of a gravid proglottid (original magnification ×40). Numerous eggs densely occupy the proglottid, making the lateral uterine branches difficult to distinguish.

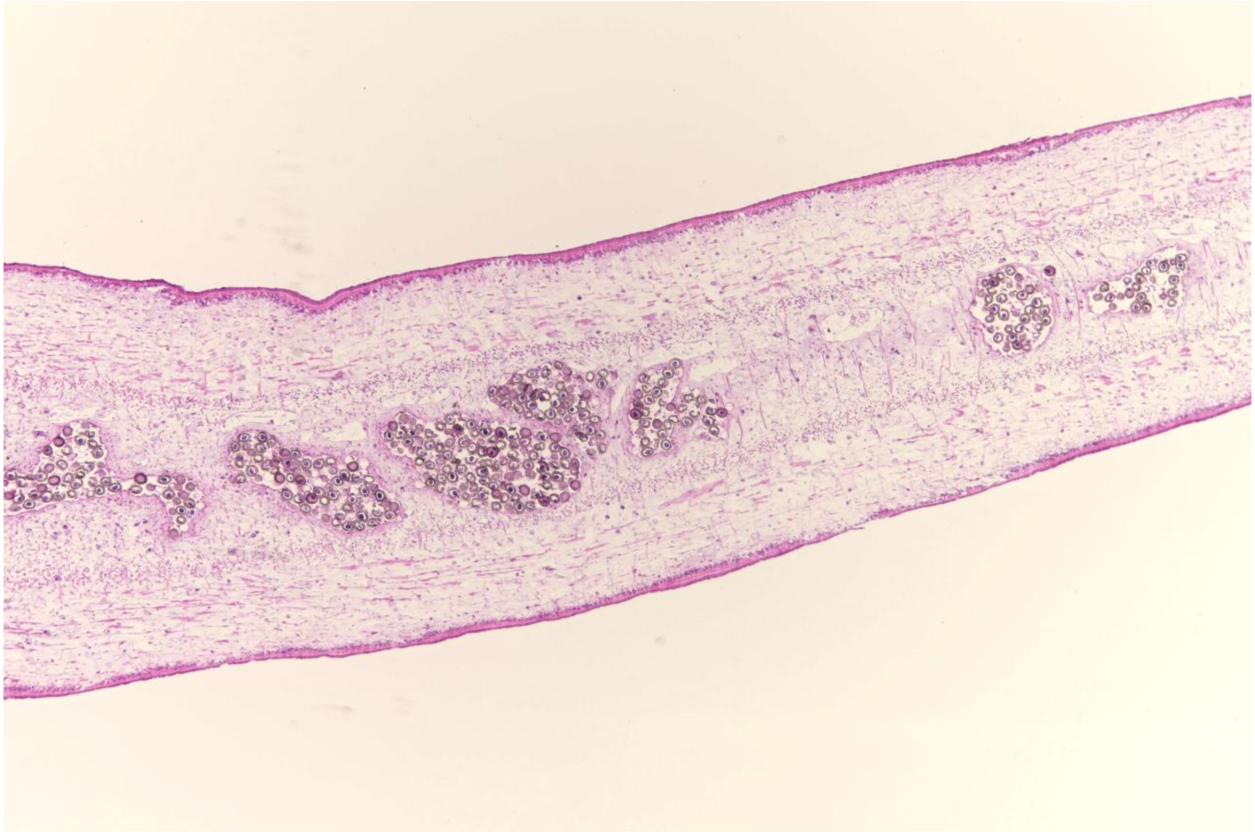

Supplement: Supplemental Materials [file tpmd250742.SD1.pdf]
